# Evaluation of patient involvement in a systematic review and meta-analysis of individual patient data in cervical cancer treatment

**DOI:** 10.1186/2046-4053-1-23

**Published:** 2012-05-07

**Authors:** Claire L Vale, Jayne F Tierney, Nicolette Spera, Andrea Whelan, Alison Nightingale, Bec Hanley

**Affiliations:** 1 MRC Clinical Trials Unit, Aviation House, 125 Kingsway, London, WC2B 6NH, UK

**Keywords:** Public and patient involvement, Consumer involvement, Systematic review, Individual patient data, Cervical cancer

## Abstract

**Background:**

In April 2005, researchers based at the Medical Research Council Clinical Trials Unit, set out to involve women affected by cervical cancer in a systematic review and meta-analysis of individual patient data to evaluate treatments for this disease. Each of the women had previously been treated for cervical cancer. Following completion of the meta-analysis, we aimed to evaluate the process of involvement from the researcher and research partner perspective.

**Methods:**

An advisory group was first established to give advice on recruiting, supporting and involving women and led to efforts to recruit women to take part in the systematic review using different approaches. Evaluation of the process and outcomes of the partnership between the systematic reviewers and the patients, in respect to what the partnership achieved; what worked well and what were the difficulties; what was learned and the resource requirements, took place during the conduct of the meta-analysis and again after completion of the project.

**Results:**

Six women, each of whom had received treatments for cervical cancer, were recruited as Patient Research Partners and five of these women subsequently took part in a variety of activities around the systematic review. They attended progress meetings and all but one attended a meeting at which the first results of the review were presented to all collaborators and gave feedback. Three of the women also became involved in a further related research project which led to an editorial publication from the patient perspective and also participated, along with two lead researchers, in the evaluation of the process and outcomes. While they were generally positive about the experience, one Patient Research Partner questioned the extent of the impact patients could make to the systematic review process.

**Conclusions:**

In general, researchers and patient research partners felt that they had learned a lot from the process and considered it to have been a positive experience. The researchers felt that because of resource implications, patient involvement in future systematic reviews would probably have to be prioritized to those in which the greatest impacts could be achieved.

## Background

Public and patient involvement in healthcare research has been widely recognized and supported by commissioning and funding bodies in the UK [1,2] and elsewhere [3]. Moreover, involvement in systematic reviews and meta-analysis has been championed by the Cochrane Collaboration [4] for some time, largely through the Cochrane Consumer Network (http://consumers.cochrane.org/) and consumer membership of Cochrane Review Groups, with the aim of ensuring the accessibility and relevance of Cochrane systematic reviews to patients, caregivers and service users. However, there are relatively few reported case examples in the medical literature that describe or evaluate patient or public involvement in specific systematic reviews. Indeed, despite extensive literature searches, a recent narrative review of patient involvement [5] identified only seven published examples, only two of which had included a quantitative meta-analysis [6,7] of which only one formally evaluated the effects of a treatment intervention [7]. This review of patient and public involvement in systematic reviews found that public involvement had made five main contributions to reviews, including refining the scope, identifying and locating relevant studies, appraising the literature, interpretation of the review findings, and writing the reports [5].

In September 2004, we initiated a systematic review and meta-analysis of chemoradiotherapy for the treatment of women with cervical cancer which aimed to collect and re-analyze individual patient data (IPD) from all relevant, eligible randomized controlled trials (RCTs) worldwide. At that time, the available evidence suggested that survival was improved in women with cervical cancer if they received chemoradiotherapy. There were some concerns among the clinical community, however, regarding long-term side effects potentially associated with this treatment. Therefore, we aimed to evaluate not only the effect of chemoradiotherapy on survival, recurrence and spread of cervical cancer, but also on the prevalence and severity of treatment-related side effects. We were keen to involve women who had experienced treatment for cervical cancer in the project, to inform the discussion about the treatments involved and, in particular, how side effects might impact on women’s day-to-day lives post treatment. We also wanted to gain a better understanding of what might be considered acceptable in terms of side effects, assuming that a survival advantage was confirmed. In addition to involving patients in the systematic review process, we also aimed to evaluate involvement with the aim of informing the practice of patient involvement in future systematic reviews conducted by our group and others. Results of the systematic review and meta-analysis have been published elsewhere [8].

## Methods

### Establishment of a Reference Group

Initially, we set up a small Reference Group, to provide advice on the recruitment of women, provision of support and information, and on the activities they might undertake. Members of this Group included two gynecological cancer nurse specialists, the Consumer Liaison lead for the National Cancer Research Network, two experts in consumer involvement in healthcare research, the then CEO of Jo’s Trust (http://www.jostrust.org.uk ) and one former cervical cancer patient, who was already known to the CEO of Jo’s Trust and who agreed to be involved both in the Reference Group and also in the systematic review.

The first meeting of the Reference Group (April 2005) led to the development of terms of reference and a role description for patients who were to get involved (Additional file 1) and to the term “Patient Research Partners” to describe the role of the women. The group also provided feedback and comments on a detailed information folder and accompanying workshop aimed at describing systematic reviews and meta-analyses to the Patient Research Partners once involved (Additional file 2). Reference Group members also helped in recruiting Patient Research Partners, both by approaching suitable women whom they knew and through the development of a job description and advert. One further meeting of the Reference Group was held in May 2007 to discuss progress and provide feedback.

### Establishing a group of Patient Research Partners

One of the most successful methods used to recruit women to be Patient Research Partners was the use of personal approaches from individual Reference Group members. In particular, approaches by the former patient (who was also a Patient Research Partner) and the CEO of Jo’s Trust. These direct approaches or recommendations led to recruitment of three women. The other most successful route to recruiting women was by advertising the opportunity to take part through CancerVoices (http://opportunities.macmillan.org.uk/opportunities.aspx). CancerVoices opportunities are distributed to people who have been affected by cancer and who are interested in participating in research projects and in sharing their experiences. Two women were recruited via this route. Other methods of recruitment, including sending information about the opportunity to become involved to cervical or gynecological cancer support groups across England, were all unsuccessful. Six women, all of whom had been treated for cervical cancer, were therefore recruited as Patient Research Partners; however, one woman, who had advanced disease, became too unwell to attend meetings and subsequently died.

### Patient research partner participation and contribution to the IPD meta-analysis

Following the first meeting of the Patient Research Partners (October 2005) they became involved in a number of activities associated with the systematic review, including providing feedback on the detailed information folders; helping to trace contact details for trial investigators; learning about data management and analysis and contributing to regular project newsletters. Four Patient Research Partners attended the Collaborators’ Meeting (May 2006), at which the first results of the systematic review and meta-analysis were presented, along with the clinicians and statisticians who had provided trial data for inclusion in the meta-analysis. The fifth Research Partner, who was unable to attend this meeting due to family commitments, subsequently chose to have no further involvement in the project.

Interim feedback from the remaining four Patient Research Partners, both on their experiences of attending the Collaborators’ meeting as well as other aspects of the project was sought (July 2006), resulting in sections describing meta-analysis results and a glossary of terms being drafted for the information folders and to the development of a ‘key findings’ document aimed at clinicians, researchers and patients.

The Patient Research Partners remained involved throughout the period during which the main publication of the systematic review and meta-analysis was being written (2007 to 2008). They provided input into the lay summary for the Cochrane systematic review [9] as well as taking part in discussions about potential research on chemoradiotherapy side effects. It was also planned to evaluate their involvement. During this period, one of the women moved abroad and took no further part in the project. In 2009, the researchers became involved in a related research project based on a Royal College of Radiologists audit of the effects of treatment for cervical cancer, including associated side effects [10]. The three remaining Patient Research Partners agreed to write a joint editorial with the researchers, to describe these side effects from a patient perspective [11]. Throughout the course of the two related research projects, six meetings were held approximately annually, more frequently at the outset. Additional communication was largely by email.

### Final evaluation of patient involvement

In August 2010, we set out to evaluate the experience from both the Patient Research Partners and researchers perspectives. While this was not a prospectively designed qualitative research project, we hoped to learn from the experiences of the Patient Research Partners and researchers to inform our future practice. Initially the Patient Research Partners and the researchers completed a short questionnaire that included a series of open questions about reasons for involvement; expectations, perceived benefits and challenges of involvement; the impact of involvement and whether and how future meta-analyses should best involve consumers. Following completion of the questionnaires, individual answers to each question were first collated and any major themes emerging from the responses were identified. A summary report was then drafted and circulated to the respondents. Finally, a meeting was held to further discuss the emerging themes and, where necessary, to add clarity and detail to the responses obtained in the individual questionnaires. Following the meeting, the summary report was revised in light of these discussions and circulated to all to ensure that it reflected accurately the discussions that had taken place.

## Results

Five questionnaires (100 %) were completed, three from the Patient Research Partners and two from the researchers. Information from the completed questionnaires was supplemented with discussion at a follow-up evaluation meeting and with feedback obtained from four Patient Research Partners in June 2006. One Patient Research Partner, who had emigrated in 2008, did not participate in the final evaluation, but provided detailed feedback in 2006. A fifth Research Partner who withdrew from the project in April 2006 did not participate in either evaluation, and we were not able to ascertain her reasons for leaving the project.

### What were the motivations for Patient Research Partners and researchers?

#### Patient research partner involvement in systematic review and meta-analysis of IPD

In general, the researchers had wanted to find out whether it was possible to properly involve patients in a systematic review and meta-analysis based on individual patient data and to better explain the process to people. They were keen to involve patients and had tried to do so in prior projects but with limited success due to difficulties in recruiting, supporting and involving them effectively. However, for this project there was funding “that allowed us to resource involvement properly” and so the researchers had “been able to give it a lot more thought; got more prepared”. The Patient Research Partners thought the project sounded interesting. One Patient Research Partner, who had previously conducted systematic reviews as a researcher, thought that “it would be interesting to be involved from a patient perspective.”

#### Survivorship issues

Perhaps the biggest motivation was that the researchers were aware of survivorship issues for women receiving these treatments for cervical cancer and felt that “patient involvement might be really helpful in getting to grips with these issues.” The Patient Research Partners also sensed that the systematic review was aiming to answer questions about the treatments they had received; “I wanted to know that I’d had the ‘right’ treatment” and “I was pleased that someone was trying to address what I perceived as the ‘gap’ once treatment was over. There was a general lack of knowledge about late side effects and that someone seemed to be trying to do something about that felt like a good thing.”

#### Positive use of the “Cancer experience”

The Patient Research Partners were eager to use their experiences to help with the research, “I was pleased to be asked and felt that my experience could be of use as someone who had recently been affected by cancer.”

### What influence and impact did Patient Research Partners have on this research?

#### Additional piece of research/editorial on patient perspectives

Perhaps the greatest impact on the researchers of working with the Patient Research Partners was that it directly led the researchers to get involved in another research project with a greater focus on late side effects of treatment. It also motivated them to publish an editorial with the Patient Research Partners, discussing the concerns of women after treatment for cervical cancer [9]. The Patient Research Partners commented that “being able to provide some information about late effects and pulling together our thoughts on these issues and getting both in print has been a great achievement” and that, “the outcome passed my expectations, as I witnessed how the team doing the evaluation realized what information was missing and how future trials could give more detailed and long term information.”

#### Adding a viewpoint that otherwise would not have been heard

The Patient Research Partners all agreed that their main impact on the research was in adding a viewpoint that otherwise would not have been heard, and reflecting the concerns of women with cervical cancer by contributing the opinions of “the normal woman off the street.” They also felt that their involvement might help to bring about changes, such that “hopefully future trials will better collect data” on late side effects. One Patient Research Partner did express concerns about her involvement though, “For a meta-analysis where the outcome measures have already been collected, I am not sure how much difference we have really made overall.”

#### Insight into cervical cancer and its treatment

The Patient Research Partners had also helped the researchers to better understand cervical cancer and its treatment, using their experience “to bring the results of the study to life as it evidenced the experience of real people.” The researchers felt the greatest impact of involving the Patient Research Partners was the insight they gained of the impact of cervical cancer, its treatments and side effects, on the women’s day to day lives, which would not have been possible without the Patient Research Partner involvement.

### What aspects of Patient Research Partner involvement worked well?

#### Recruitment

The Patient Research Partners felt that working with charities like Jo’s Trust, which supports women with cervical cancer (or similar groups for other disease areas), was probably a very effective way to recruit patients, because the people who use them are, “very involved in their disease and its treatment and will also potentially have wider networks of people that they are in contact with to draw experiences from.” Advertising the opportunity through CancerVoices was also felt to have been an effective strategy by the researchers and Patient Research Partners, because CancerVoices’ volunteers have received training to help them to understand their role in research and on effective meeting conduct. This gives individuals more confidence in their role and what may be expected of them. All agreed that having a small group of Patient Research Partners had worked well, as the group had felt balanced in terms of the numbers of Patient Research Partners and researchers.

#### Information provision

The researchers had developed an information pack for the Patient Research Partners (Additional file 2), which was supported by presentations and discussions about the project at meetings. On the whole, the Patient Research Partners had found this useful and felt it was easy to understand, providing them with the information they needed without overloading them. “The presentations and design of the materials took great care to unravel the complex world of research acronyms and concepts and explain complex ideas simply but without dumbing down. That made me feel that we were equal partners in a really important piece of work.”

#### Being of value

One of the key things from the Patient Research Partners perspective was being made to feel that their contribution was valued. One Patient Research Partner said “It was also really pleasing that the hard work put into the research project was recognized by the research community and published. It’s a good feeling from a patient perspective to have contributed to a piece of work which recognizes the after effects of treatment and survivorship issues.”

#### Collaboration of researchers and patients

The Patient Research Partners said that overall they had enjoyed being part of the team, learning about and gaining insight into the research process, “We were part of a shared experience of the scientific and patient communities working together to produce these outcomes, so it felt like a collaborative venture. Quite often as a patient you feel second to the might of medicine and science and it was really good to feel that our experience was important and could help shape a piece of work in an area where there was little info.”

Although there were some mixed feelings about their participation at the Collaborators’ Meeting (see also section What aspects of Patient Research Partner involvement didn’t go so well?), at which results were presented to the collaborative group for the first time, the Patient Research Partners had mostly appreciated the opportunity to attend, “the conference with senior practitioners from all over the world was a very memorable experience for me. Understanding their priorities and their perspective on cancer treatment was very revealing and made the work with Patient Research Partners all the more essential to help build a holistic picture of people living with cancer and their needs.”

The Researchers felt that they learned a lot from the Research Partners and in doing so, had overcome their initial concerns and anxieties about involving Patient Research Partners, such as being tokenistic and also about discussing potentially “taboo” or sensitive subjects, “We aren’t medical or nursing professionals so don’t have experience of working with patients. We had no idea how well or poorly the women were going to be or whether any of them were coming into it with preconceptions that would make it difficult to work with them.” The researchers felt that they had established good working relationships with the Patient Research Partners and together, had achieved a lot.

### What aspects of Patient Research Partner involvement didn’t go so well?

#### It took a long time

One concern raised by the Patient Research Partners was the length of time the project had taken. “The length of time the research took was quite long, however I understood that collating the information was not an easy task, particularly when trying to locate trials in other countries and getting the information from them”. One of the key issues this raised was that as time passed, understandably, one of the Patient Research Partners said that her “personal interest in cancer has faded somewhat.”

#### Informed choices

Some comments and discussions at the Collaborators’ meeting had been difficult or upsetting for the Patient Research Partners, “In the lead up to the Collaborators meeting it would have been good to have been better informed about the sorts of discussions that would take place…that the clinicians are going to be blunt and scientific in their approaches and not the normal ’bedside manner’ we might be used to as patients!” All agreed that it would be important to provide Patient Research Partners with more information about the potential nature of discussions at the meeting to ensure that they had the ability to make an informed choice about attending the meeting and also to ensure that the clinicians attending the meeting knew that they were patients. Furthermore, the Patient Research Partners felt that there had been insufficient preparation regarding what the results of the meta-analysis would look like. This feedback led to a section being drafted and added to the information pack, with the input of the Patient Research Partners, to explain how meta-analysis results are presented and what they mean.

#### Useful input?

Interestingly, one of the Patient Research Partners expressed that she had concerns at the outset regarding how much input ‘users’ could really make in the context of a systematic review, given that the outcomes are to a degree “pre-set by the outcomes that were collected within the individual trials”. In this study, the protocol development took place prior to Patient Research Partners becoming involved, so it was felt that there would be more opportunity for input if Patient Research Partners had been involved earlier in the project. However, although the review aimed to evaluate treatment side effects, it was not possible as suitable data were not collected in all of the trials. One of the researchers expressed that this had been disappointing, because it “felt like we’d perhaps got the Research Partners involved under false pretences and worried about what they would get out of it because of the lack of data. With hindsight we were perhaps overly optimistic about what we could do.”

#### Resource implications

For this project, funding received through the UK Department of Health (DoH) NCCRCD Evidence Synthesis award scheme provided the resources to hold meetings, pay the Research Partners for their time and reimburse expenses. The researchers felt that this was important as involving the Patient Research Partners had taken extra time and effort; for example, the information pack had taken some time to develop and produce. However, since the information pack developed with the Research Partners will form a template, similar information for future projects may take considerably less time. The pressure on researchers’ time only became an issue around key points of the meta-analysis, “It was sometimes difficult to bridge the needs and priorities of the Patient Research Partners and the clinical/scientific collaborators.” In addition, using the experience gained through this project, the researchers felt that there would be less need for an extensive Reference Group in subsequent projects, further reducing the resource requirement. Furthermore, because the group was already established and used to working together, it had been very easy to involve them in the subsequent work around the drafting of the editorial.

### What was learned about involving patients in future IPDMAs?

#### Opportunity for real input

All of the Patient Research Partners said they would consider getting involved in research again in the future. “It was my first experience of user involvement and I would certainly do it again if I felt that my experience was relevant and could usefully contribute to research.” However, one of the Patient Research Partners doubted that she would get involved in another systematic review, as she felt that there is more opportunity to shape primary research. In future reviews, researchers felt that Patient Research Partners should be involved at the stage when the questions are being developed to give them the opportunity to influence which outcomes should be investigated. The researchers felt that they could only commit to a similar approach to patient involvement for systematic reviews based on IPD where there is resource and perhaps as importantly, time, to train, support and develop working relationships with patients. Other potentially less resource-intensive models to involvement could be adopted for standard systematic reviews. Also, to ensure that Patient Research Partners were truly able to influence the research, it was felt that involvement should be prioritized for projects in which the outcomes may be of most interest to patients and where patient input might have the greatest impact on the research, for example, survivorship issues, patient centered outcomes or quality of life.

#### Being selective about recruitment

The researchers were concerned about what they would do if they received too many responses from interested patients. One Patient Research Partner suggested that at the outset, patients interested in becoming involved could “attend an information session or meeting to see what the project was about and understand the commitment and what their role would be” before deciding to participate. Patient Research Partners also thought it would be appropriate for the researchers “to be selective and only include people who can bring something to the project.” All were in agreement that a small group (three to five members) of Patient Research Partners had worked well in this review and would be a good model for the future. They also were enthusiastic about the idea of mentors being provided for future Patient Research Partners; for example, Patient Research Partners who had already been involved in systematic reviews.

#### Improved preparations for patient involvement

To overcome some of the difficulties experienced in this review, future projects would need to set out realistic time-lines at the outset, better inform the Patient Research Partners about any delays to progress of the project, provide better information about how the results of meta-analyses are presented, and to better explain to the Patient Research Partners about the format of the Collaborators’ meeting, giving them the opportunity to opt in or out.

## Discussion

Our results describe the benefits and challenges of researchers and patients carrying out systematic reviews in partnership. The Patient Research Partners were involved in three of the five main areas identified by Boote and colleagues [5], namely, locating study investigators, interpretation of the results from the patient perspective and writing an editorial on the findings of the review and related research. We acknowledge that it would have been preferable to involve patients at an earlier stage to enable them to help define the scope of the systematic review and would aim for this to be the case for future meta-analyses of IPD conducted within our group. While the Patient Research Partners were not involved in appraising the studies identified for inclusion, we tried to ensure that the reasons for collecting and reanalyzing IPD in this review were well understood and two of the Research Partners observed the process of checking and managing incoming trial data.

Results of this evaluation are potentially limited in that only three Patient Research Partners and two researchers contributed to it. Also, there is a possibility that individuals may have held back some of their more negative comments or concerns because the discussions were open to all. However, the discussions were frank and honest and the concerns of those involved were discussed fully. Furthermore, we cannot be certain how the feelings of the Patient Research Partner who withdrew from the project might have impacted on our results as she did not complete an evaluation or provide her reasons for withdrawing. Nevertheless, both the researchers and Patient Research Partners thought that involvement had been rewarding and worthwhile and all involved felt they had learned from the experience. Good information had been vital in helping the Patient Research Partners to gain an understanding of the review and regular communications were important in maintaining their interest throughout the project. However, the project took a long time to complete and with the benefit of hindsight, they could have been better warned about this in advance. All of those involved felt that they would consider involvement in future research projects; however, one Research Partner was uncertain about involvement in further systematic reviews because of the time frame and the potentially limited influence on the research. However, despite this, highlighting gaps in the evidence through a systematic review was felt by all to be worthwhile.

In our setting of gynecological cancer, we were aware that some of the issues were highly sensitive and, therefore, potentially difficult to discuss. Furthermore, for recently treated patients, there is the possibility that the disease could return or spread, making further involvement either practically or emotionally difficult, which researchers may need to consider and plan for in advance. Different issues or challenges may arise for reviews in different settings; for example, when working with parents of sick children, people with mental health problems or those with acute illness.

One of the benefits of systematic reviews using IPD taking considerable time to complete is that researchers and patients had time to develop good working relationships and to gain an understanding of the process and purpose of the research. Involvement of patients in this systematic review and meta-analysis undoubtedly used additional time and resources, which may be problematic where reviews need to be conducted in a limited timeframe or on restricted budgets. For standard systematic reviews using published results, where there may be less opportunity for Patient Research Partners to shape the research and more limited time in which to complete the review, other approaches to involvement may be more appropriate or more realistic; for example, involving people with prior knowledge of the disease and/or systematic reviews or engaging with established patient groups and organizations. Alternatively, it may be necessary to prioritize involvement in those projects most likely to benefit from the input of patients; for example, projects in which long-term side effects or quality of life outcomes are key issues or in chronic or life-changing diseases. In this project, specific funding allowed for the payment of research partners, reimbursement of travel and other expenses for meeting attendance and other overheads associated and importantly, enabled researchers’ time to be spent on involvement activities. We would strongly recommend that researchers planning to involve patients in their research request additional resources in funding applications.

Interestingly, Patient Research Partners were comfortable with the idea that researchers should be selective about involvement. They felt that recruiting patients who were in a good position to bring skills or ideas to the group was acceptable and also understood that it might be appropriate for researchers to consider what consumer involvement might add to a specific systematic review and to target involvement appropriately. While it was felt that it had been quite resource intensive to establish a small group of consumers to participate in the review, once established, this model has a number of advantages. For example, access to varied opinions, enabling almost immediate involvement in follow-up or closely related research projects and also the possibility of mentoring or helping in the development of individuals in new research projects.

In the early stages of this project, there was little guidance available to researchers on involving patients in systematic reviews. This meant that the Reference Group was vital to facilitate patient involvement and to advise the researchers. A similar model may benefit researchers coming to patient involvement for the first time. Furthermore, it may be helpful to seek advice from other researchers or groups with experience of involving patients. New guidance, led by a group of researchers and patients on behalf of INVOLVE (http://www.INVO.org.uk), on patient and public involvement in systematic reviews, is currently in development and, once available, should help those embarking on involving patients. We would certainly recommend that reviewers also try to engage with a relevant patient group or organization when embarking on a new review.

A recent review of published examples of patient involvement in systematic reviews [5] found only seven relevant examples across a broad spectrum of health and social care research. Therefore, our evaluation provides one of very few examples in the literature to date of patient involvement in a systematic review and meta-analysis, and perhaps the only example of involvement in a systematic review based on individual patient data. We would encourage reviewers who have involved patients and the public in their reviews to report their experiences in order that others may apply the lessons learned by others in their own reviews.

## Conclusions

This evaluation of patient involvement in a systematic review and meta-analysis of individual patient data has shown that patient involvement is possible and may benefit the review process. Both the researchers and Patient Research Partners found it to be a positive experience. It does, however, require additional resources and careful consideration should be given at the outset regarding the reasons for involvement and its potential impact on the individual review. A small group approach had a number of advantages in this review; however, other approaches to involvement may suit different types of review. Researchers carrying out systematic reviews and involving patients or the public in their reviews are encouraged to publish their findings so that others may learn from their shared experiences and to expand the evidence base for involvement in reviews.

CEO, chief executive officer; DoH, Department of Health (UK); IPD, individual patient data; NCCRCD, National Coordinating Centre for Research Capacity Development; RCTs, randomized controlled trials

## Competing interests

None of the authors have any competing interests to declare with regard to this work.

## Authors’ contributions

CV, JT and BH had the original idea to conduct this evaluation. CV developed the questionnaire and arranged the meetings that were held with the Patient Research Partners. JT, CV, AW, AN and NS completed the evaluation questionnaires and attended the discussion meeting, which was chaired by BH. CV collated the responses and drafted the manuscript with the help of all authors. All authors read and approved the final manuscript.

## Author information

CV and JT are part of the Meta-Analysis Group at the MRC Clinical Trials Unit. They have both been responsible for carrying out systematic reviews and meta-analysis, mostly in cancer, over a number of years and have an interest in patient and public involvement in meta-analyses. BH is a co-director of TwoCan Associates, and has a particular interest in clinical trials. She works as an adviser to the MRC CTU on patient and public involvement. She has undertaken and published research on patient involvement in clinical trials. AN, AW and NS are the Patient Research Partners. They come from different walks of life and different areas of the UK. They have all received treatment for cervical cancer and have volunteered to be involved in the meta-analysis as part of the Patient Research Partners’ group.

## Supplementary Material

Additional file 1 Terms of reference and Role Description for Patient Research Partners. Click here for file

Additional file 2 Detailed information on the systematic review and meta-analysis of individual patient data in cervical cancer. Click here for file
