# Supplementary material for: Evaluation of patient involvement in a systematic review and meta-analysis of individual patient data in cervical cancer treatment
Source: Syst Rev. 2012 May 7;1:23. doi: 10.1186/2046-4053-1-23 (PMC3407735; doi:10.1186/2046-4053-1-23)
Supplement: Additional file 1 — Terms of reference and Role Description for Patient Research Partners. [file 2046-4053-1-23-S1.doc]

# Research Partners: Terms of Reference

The Terms of Reference should act as a guide for the Research Partners. They should give you a better idea about the your role in this project if you become a Research Partner. They explain what we would expect from you if you became a Research Partner, as well as what you can expect from us. The Terms of Reference can change in response to your feedback.

## Membership

We hope to set up a group of 5-6 Research Partners to be involved in the project. The two requirements that we think Research Partners need are:

1. Personal experience of either radiotherapy or chemoradiation treatment for cervical cancer
2. Interest and enthusiasm about being involved in this project

You do not need to have any previous experience of research. And, you are free to leave at any time, although we hope that all Research Partners would like to be involved throughout the project. We expect that this project will take around 2 years to complete.

## What we can provide to Research Partners

If you decide to become a Research Partner, we are committed to providing you with:

- An opportunity to learn about, actively contribute to and influence research
- Flexibility and choice in your level of involvement in the project
- A safe and supportive research environment
- Appropriate and relevant training
- A mentor, to act as your first point of contact for queries or difficulties
- Opportunities to meet other Research Partners to discuss the project
- Payment for attending meetings where needed

We will work with you to:

- Help you to learn about and understand the research project
- Identify research and/or administrative tasks that you can help with
- Provide access to appropriate training, where it might be helpful
- Support you in the activities that you undertake

Claire Vale, Meta-analysis Group, MRC Clinical Trials Unit, London April 2005
IPD meta-analysis of chemoradiation in cervical cancer

We will provide a safe and supportive environment for your involvement, but the Research Partners group is not intended to be a Support Group. We are not able to give specific information or advice on treatments that you might have been given or may be receiving. However, we can put you in touch with groups or individuals who can provide emotional support and advice, should you want this.

## Key Responsibilities and Aims

As a Research Partner, you will work with each other and members of the Meta-analysis Group to carry out the research project. You will also feedback your thoughts and experiences of being involved in the project to the Meta-analysis Group and Reference Group. We would also like two Research Partners to be members of the project Reference Group. This responsibility could easily be shared between the Research Partners. There are separate Terms of Reference available for the Reference Group members.

There are many aspects to running a research project like this one. Some of these are administrative or creative and not necessarily scientific or technical. We can discuss the possibilities with you to help you to find things that interest you and that you might like to be involved with. For example there may be opportunities to:

- Read and comment on the information that we produce about the project for women who have had or have cervical cancer
- Help us to organise small local meetings or the large Collaborators’ meeting at the end of the project
- Write short articles for the newsletter
- Help to produce and disseminate the results of the project to women who have had or have cervical cancer

You can be involved in one or more of the many aspects of this project. What you do might change as the project progresses. For example, once you have a clearer idea of the project, and have maybe taken up opportunities for training, you might take on different tasks or suggest other areas where your skills could put to good use. We will work with you to develop these opportunities.

Claire Vale, Meta-analysis Group, MRC Clinical Trials Unit, London April 2005
IPD meta-analysis of chemoradiation in cervical cancer

## Meetings and Communications

We can arrange to visit you or a have a telephone conversation with you so that you can find out more about project before you decide whether or not to become a Research Partner. Once 5 or 6 women have agreed to be Research Partners, we will organise a meeting at the MRC Clinical Trials Unit, or at another convenient location. We will try to keep this meeting (and any additional meetings we have) very informal and we won’t use technical language or jargon. This meeting will give you:

- Chance to meet the other Research Partners
- Chance to meet the Meta-analysis Group members who are running this project
- Opportunity to find out more about the project and decide if you would like to be involved in it

After this first meeting, we will contact you in the way you prefer, and we can send you information in whichever format suits you best (e.g. on paper, email, large print etc). If you need to ask any questions or if you want us to explain something, you can contact us during our working hours to ask. We will do our best to answer all enquiries promptly and clearly. We might also organise other meetings of the Research Partners during the project, but we would like to keep the number of meetings to a minimum. We will try to arrange them to suit the needs of the individuals.

## Payment

Where needed, we can reimburse you for travel or other expenses for attending meetings. These might include overnight accommodation or carer costs, for example. Wherever possible, we will try to pay for all travel tickets and accommodation bookings in advance. Otherwise, we will aim to reimburse expenses as quickly as possible. In addition, we are able to pay Research Partners for their time at the standard Department of Health rate (currently £138.71/day).

Claire Vale, Meta-analysis Group, MRC Clinical Trials Unit, London April 2005
IPD meta-analysis of chemoradiation in cervical cancer

## Person Specification

| **Attributes** | **Essential** | **Desirable** | **Not  important** |
| --- | --- | --- | --- |
| Enthusiastic about the project | ✓ |  |  |
| Committee Experience |  |  | ✓ |
| Aware of issues that might affect women with cervical cancer |  | ✓ |  |
| Understanding of medical / research language |  |  | ✓ |
| Experience of chemoradiation or radiotherapy treatment for cervical cancer | ✓ |  |  |
| Good communicator /able to express own views in a mixed group of professionals and consumers |  | ✓ |  |

Claire Vale, Meta-analysis Group, MRC Clinical Trials Unit, London April 2005
IPD meta-analysis of chemoradiation in cervical cancer
