# Supplementary material for: Evaluation of patient involvement in a systematic review and meta-analysis of individual patient data in cervical cancer treatment
Source: Syst Rev. 2012 May 7;1:23. doi: 10.1186/2046-4053-1-23 (PMC3407735; doi:10.1186/2046-4053-1-23)
Supplement: Additional file 2 — Detailed information on the systematic review and meta-analysis of individual patient data in cervical cancer. [file 2046-4053-1-23-S2.doc]

# Contents

[**Contents 1**](#__RefHeading___Toc188779776)

[Introduction 1](#__RefHeading___Toc188779777)

[The Meta-analysis Group 1](#__RefHeading___Toc188779778)

[About this folder 2](#__RefHeading___Toc188779779)

[What is cervical cancer? 3](#__RefHeading___Toc188779780)

[The cervix 3](#__RefHeading___Toc188779781)

[Cancer of the cervix 3](#__RefHeading___Toc188779782)

[Stages of cervical cancer 4](#__RefHeading___Toc188779783)

[Early cervical cancer 4](#__RefHeading___Toc188779784)

[Locally advanced cervical cancer 4](#__RefHeading___Toc188779785)

[Advanced cervical cancer 5](#__RefHeading___Toc188779786)

[Treatments used in cancer 6](#__RefHeading___Toc188779787)

[What treatments do women with cervical cancer get? 7](#__RefHeading___Toc188779788)

[Surgery for cervical cancer 7](#__RefHeading___Toc188779789)

[Radiotherapy for cervical cancer 8](#__RefHeading___Toc188779790)

[Chemoradiation for cervical cancer 8](#__RefHeading___Toc188779791)

[What are clinical trials? 10](#__RefHeading___Toc188779792)

[Randomised controlled trials 10](#__RefHeading___Toc188779793)

[Why do we need systematic reviews and meta-analyses? 11](#__RefHeading___Toc188779794)

[What is a systematic review and a meta-analysis? 12](#__RefHeading___Toc188779795)

[What is an individual patient data meta-analysis? 13](#__RefHeading___Toc188779796)

[What do we already know about chemoradiation in cervical cancer? 14](#__RefHeading___Toc188779797)

[What might this project tell us? 15](#__RefHeading___Toc188779798)

[Understanding the results of a meta-analysis 16](#__RefHeading___Toc188779799)

[What information do we collect on each woman? 16](#__RefHeading___Toc188779800)

[What data do we collect on the trial? 19](#__RefHeading___Toc188779801)

[How do we analyse the results? 20](#__RefHeading___Toc188779802)

[How do we display meta-analysis results? 21](#__RefHeading___Toc188779803)

[Example of a forest plot 26](#__RefHeading___Toc188779804)

[Being a Research Partner 27](#__RefHeading___Toc188779805)

[Why involve Research Partners? 27](#__RefHeading___Toc188779806)

[Conclusions 28](#__RefHeading___Toc188779807)

[Notes 29](#__RefHeading___Toc188779808)

[Feedback Form 31](#__RefHeading___Toc188779809)

[Research Partners: Draft Terms of Reference 33](#__RefHeading___Toc188779810)

[Membership 33](#__RefHeading___Toc188779811)

[What we can provide to Research Partners 33](#__RefHeading___Toc188779812)

[Key Responsibilities and Aims 34](#__RefHeading___Toc188779813)

[Meetings and Communications 35](#__RefHeading___Toc188779814)

[Payment 35](#__RefHeading___Toc188779815)

[Person Specification 36](#__RefHeading___Toc188779816)

[Glossary 37](#__RefHeading___Toc188779817)

# Introduction

## The Meta-analysis Group

Our group is the Meta-analysis Group of the Medical Research Council Clinical Trials Unit in London. We are a group of five researchers who specialise in this type of project. In the past, we have done meta-analyses in different types of cancer including ovarian cancer, lung cancer, oesophageal and cervical cancer.


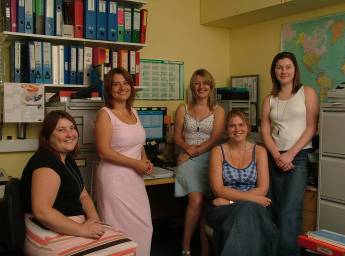


**The meta-analysis group**

In 2003, we began to organise this meta-analysis project, which is trying to find out more about giving women with cervical cancer radiotherapy and chemotherapy at the same time (chemoradiation). Since then we have done the preparations and planning stages of the research. The project involves many doctors and researchers who are interested in cervical cancer.

In December 2004, we began to look at ways to involve women who had a personal experience of cervical cancer as Research Partners in this project. This was because at this time, Claire got awarded some funding through the Department of Health, which meant that we could aim to do this in a meaningful way. It also means that we able to pay these women for their time.

## About this folder

We would like to involve some women who have been treated with either radiotherapy or chemoradiation for cervical cancer as Research Partners in this project. At the back of the folder there are Terms of Reference for the Research Partners. If you are interested in getting involved in the research project, please have a look at these. You can also contact us to find out more about being a Research Partner.

This information in this folder aims to help to explain the project in a way that everyone can understand. We have tried not to use any 'jargon', but if we have had to use it, we have tried to explain it, so that it makes sense. We hope that you find this information interesting and helpful. We have also supplied two booklets that are produced by CancerBacup, called Understanding Cancer of the Cervix and Understanding Radiotherapy. These booklets provide more information about cervical cancer and its treatment than we have written for this folder.

If there is any other information that you think might be helpful, please let us know and we can try to provide it for you. There is also a section where you can make your own notes. You might like to add some questions of your own that we will try to help you to answer.

If you have any questions or feedback, or if you would like to be involved in the project, please contact us at the address below.

Claire Vale

Meta-analysis Group

MRC Clinical Trials Unit

222 Euston Road

London NW1 2DA

E-mail: cv@ctu.mrc.ac.uk

Telephone: 020 7670 4723

# What is cervical cancer?

## The cervix

The cervix, along with the womb, vagina and the ovaries make up a woman's reproductive system. The cervix is the opening to the womb from the vagina.

**The female reproductive system with a close-up of the cervix**


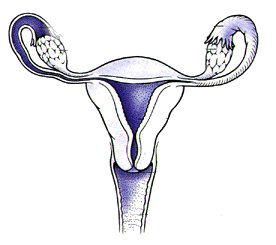


**(To the vagina)**


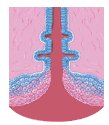


**Passageway from the cervix to the womb**

**(To the womb)**

**Cervix**

**Vagina**

**Cervix**

**Womb**

**(uterus)**

**}Ovaries**

## Cancer of the cervix

There are two main types of cancer of the cervix. They are named after the type of cells that become cancerous. Squamous cell carcinoma starts in the skin-like cells that cover the outside of the cervix. Adenocarcinoma starts in gland cells that are found in the passageway from the cervix to the womb. Both types of cancer are treated in the same way.

# Stages of cervical cancer

Doctors use a system for cervical cancer that numbers the different stages from 0 to 4. The stage of a cancer describes how big the tumour is and how far it has spread.

## Early cervical cancer

**Stage 0** - there are cancerous cells at the surface of the cervix, but they have not spread anywhere. Sometimes doctors call stage 0 “Carcinoma in situ”

**Stage 1a** - the tumour can be only be seen using microscope and is still just in the cervix. It has not spread by any more than 5 millimetres (mm) deep or 7 mm across.

Stage 1a1 means the tumour is no more than 3 mm deep in the tissues of the cervix.

Stage 1a2 means the tumour has spread between 3 and 5 mm deep in the tissues of the cervix.

**Stage 1b** the cancer is larger and can be seen with the naked eye. It has not spread anywhere else and so it is still only in the cervix.

Stage 1b1 means the tumour is up to 4 centimetres (cm) across

## Locally advanced cervical cancer

Stage 1b2 means the tumour is bigger than 4cm across. This stage is sometimes called “bulky stage 1b.” Because the tumour is bigger, doctors treat stage 1b2 as if it were locally advanced cervical cancer.

**Stage 2** - the tumour has begun to spread to the tissues next to the cervix.

Stage 2a means that the tumour has spread down into the upper part of the vagina.

Stage 2b means that the tumour has spread sideways, into the tissue next to the cervix. This tissue is called the parametrium.

**Stage 3** – means the tumour has spread further away from the cervix.

Stage 3a means the tumour has spread further down into the lower part of the vagina, but it has not spread sideways

Stage 3b means that the tumour has spread sideways on to the pelvic wall. It can also mean that the tumour is causing kidney problems, for example, the kidney is swollen because the tumour is blocking tubes that lead from the kidneys that urine is passed through (a condition known as hydronephrosis)

**Stage 4** - the tumour has spread into other body organs.

Stage 4a the tumour has spread into organs nearby the cervix such as the bladder or the rectum

## Advanced cervical cancer

Stage 4b cervical cancer, the cancer has spread to organs that are further away from the cervix, such as the lungs.

# Treatments used in cancer


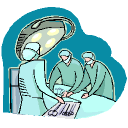
Most people who have cancer are treated either with surgery, radiotherapy, chemotherapy or a combination of two or more of these. Newer treatments such as antibody treatments or hormone treatments may also be available for some cancers.

**Surgery** is done to remove the tumour. Doctors will sometimes have to remove the tissues or organs around the tumour as well. This is to try to make sure that all of the cancer has been removed.


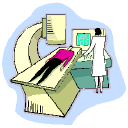
**Radiotherapy** is where x-rays are used to try to kill the cancer cells. Radiotherapy can be given from outside the body (external radiotherapy) or from inside the body (internal radiotherapy or brachytherapy).

**
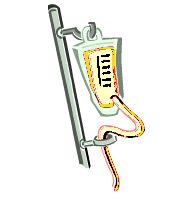
**

**Chemotherapy** is where drugs are used to try to kill cancer cells. Most often, the chemotherapy is given through a drip into a vein. Sometimes doctors use chemotherapy on its own or with either surgery or radiotherapy. If chemotherapy is given first (*before* surgery or radiotherapy), it is called neoadjuvant chemotherapy. Doctors think that neoadjuvant chemotherapy might help to shrink the tumour, which could make it easier to remove it with surgery or kill it with radiotherapy. Chemotherapy given *after* surgery or radiotherapy is known as adjuvant chemotherapy. Doctors use adjuvant chemotherapy to try to kill any cancer cells that might have been left in the body after surgery or radiotherapy. In some cancers, doctors may give chemotherapy and radiotherapy *at the same time*. This treatment is called concomitant or concurrent chemoradiation (or chemoradiotherapy).

# What treatments do women with cervical cancer get?

Women with cervical cancer are treated with surgery, radiotherapy, chemotherapy or a combination of these treatments. Doctors have to decide what treatment to offer women with cervical cancer. What they offer will depend on:

- the stage of cancer that the woman has
- her age
- how fit she is
- whether she is overweight
- whether she may want to have children in the future
- her preference

Therefore, the most appropriate treatment might be different for each woman.

## Surgery for cervical cancer

There are different types of surgery that can be used to treat women with cervical cancer.

**Cone biopsy** is where the surgeon removes a cone-shaped area of tissue that includes the tumour and a small amount of the tissue around it. Cone biopsy is only suitable for women with stage Ia1 cervical cancer.

**Radical trachelectomy** is a newer type of operation for cervical cancer. The surgeon removes the cervix, the upper part of the vagina, the tissue around the lower part of the womb (the parametrium) and the pelvic lymph nodes. However, the womb and the ovaries are not removed. This means that it may be possible for women treated with radical trachelectomy have children. Radical trachelectomy is only considered for women with stage Ia1, 1a2 or small stage 1b1 cervical cancer who may want to have children in the future. Because it is a fairly new operation, it is not widely available in all counties.

**Simple hysterectomy** is an operation where the surgeon removes the cervix along with the womb and fallopian tubes. The surgeon may also take out some of the pelvic lymph nodes, to find out if the cancer has spread. Women who have stage Ia1 or Ia2 cervical cancer may be treated with a simple hysterectomy. Women treated with simple hysterectomy will not be able to have children after their treatment.

**Radical hysterectomy** is a much bigger and more complicated operation than a simple hysterectomy. The surgeon removes the cervix, womb, the tissue around the lower part of the womb (the parametrium), the fallopian tubes, the top of the vagina and the tissue alongside the cervix. They also take out the pelvic lymph nodes, to see if the cancer has spread. Women who have stage Ib1 or stage IIa cervical cancer may be treated with a radical hysterectomy. Women treated with radical hysterectomy will not be able to have children after their treatment.

## Radiotherapy for cervical cancer

Women with cervical cancer are often treated using **radical radiotherapy.** In cervical cancer, radical radiotherapy is given:

- Externally – by machines outside the body and
- Internally – by putting a radioactive source into the vagina and womb. This type of internal radiotherapy is sometimes called brachytherapy.

Radical radiotherapy may be used to treat women who have stage Ib1, IIb, IIIa, IIIb and IVa cervical cancer. Like simple and radical hysterectomy, women treated with radical radiotherapy will not be able to have children after their treatment.

## Chemoradiation for cervical cancer

In recent years, some doctors have started to treat women with cervical cancer with **chemoradiation**. This is where chemotherapy and radiotherapy are given at the same time. Chemoradiation may be used to treat women with stage Ib1, Ib2 (bulky stage Ib), IIa, IIb, IIIa, IIIb and IVa cervical cancer.

In this project, we are looking closely at chemoradiation. The information in this folder explains why many people now think that chemoradiation is better than radiotherapy or surgery alone. It also explains what we hope this project will let us find out.

The treatments described above and summarised on the table on the next page are the usual treatment options for women with early and locally advanced cervical cancer. Some women may be offered other ‘new’ or ‘experimental’ treatments. This will usually be as part of a clinical trial that her doctor is involved in.

### Table 1. Choices of treatment for women with cervical cancer

| **Stage of  cervical cancer**  **Early** **⭢ Locally advanced** | **Choice of treatment** | | | | | |
| --- | --- | --- | --- | --- | --- | --- |
| **Stage 0** | **Cone biopsy** |  |  |  |  |  |
| **Stage 1a1** | **Cone biopsy** | **Radical trachelectomy** | **Simple hysterectomy** |  |  |  |
| **Stage 1a2** |  | **Radical trachelectomy** | **Simple hysterectomy** |  |  |  |
| **Stage 1b1** |  | **Radical trachelectomy** |  | **Radical hysterectomy** | **Radical radiotherapy** | **Chemoradiation** |
| **Stage 1b2** |  |  |  |  |  | **Chemoradiation** |
| **Stage 2a** |  |  |  | **Radical hysterectomy** |  | **Chemoradiation** |
| **Stage 2b** |  |  |  |  | **Radical radiotherapy** | **Chemoradiation** |
| **Stage 3** |  |  |  |  | **Radical radiotherapy** | **Chemoradiation** |
| **Stage 4a** |  |  |  |  | **Radical radiotherapy** | **Chemoradiation** |

# What are clinical trials?

Clinical trials are research studies that involve patients. Doctors use clinical trials to try to find out if a new treatment looks promising or to find out how best to give the treatment to patients. Some clinical trials are done to find out if a new treatment has any unexpected or unpleasant side effects. These types of trials are usually fairly small.

## Randomised controlled trials

The type of clinical trial that we are interested in is a randomised controlled trial. In randomised controlled trials, one treatment is compared with another. They are needed when doctors aren't sure which one of the treatments is better.

In a randomised controlled trial, a new treatment is usually compared with the best treatment that is already being used (the control). One group of patients in the trial is treated with the new treatment while the other group receives the control.

It is really important that the groups are broadly similar with a good mix of patients in each of the two groups. This is done using a process called randomisation, which determines which treatment each patient will be given.

When a patient decides to take part in a randomised trial, neither they nor their doctor knows beforehand which of the treatments they will get. Because who gets which treatment cannot be influenced by the doctor (or the patient) and because the process of randomisation creates groups of patients that are similar, then the treatments can be compared fairly at the end of the trial. Doctors can be fairly confident that any differences (for example, a difference in survival) are because of the treatments and not because the two groups of patients are different.
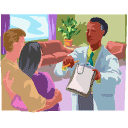
 The results of randomised controlled trials are therefore thought to be very reliable.

# Why do we need systematic reviews and meta-analyses?

Individual randomised controlled trials aren’t always able to answer questions that we ask. This might be for a number of reasons, for example if the difference in the effect between the new treatment and the control is very small. Even a small difference might be important to patients, but small differences are very hard to spot. Randomised controlled trials might need many hundreds or even thousands of patients to spot these differences reliably. However, it can be very difficult to recruit enough patients to take part in a trial. If a trial has too few participants, then doctors cannot be sure whether any differences they see are because of the different treatments, or just because of chance. This is why it can sometimes seem as if trials give different answers.

Randomised controlled trials that have asked the same questions might seem to give different answers about the effect of a treatment from one another. Because of this, it is really important to look at the results from all of the trials that have been done, so that you can get a balanced picture. It’s a bit like a jury being able to study all of the evidence in a court case before they make their decision.

# What is a systematic review and a meta-analysis?

Systematic reviews and meta-analyses are all about looking at all of the evidence. All the trials that have asked the same question can be looked for (a systematic review) and their results combined together (a meta-analysis).


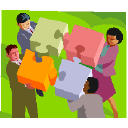
It’s a bit like doing a jigsaw puzzle where each of the trials is a piece of the puzzle. A systematic review is like gathering together all of the pieces that you need to make the picture. A meta-analysis is putting all of the pieces together to make the picture. If some of the trials are missing, you don’t get the full picture. Having all of the trials means that you get a much clearer picture. And having many more patients means that you can spot small differences between the treatments much more reliably. If a systematic review is not done, it is like doing a jigsaw that has pieces missing. You can never be sure that you have got all of the trials and so you won't know how complete or reliable the answer is.

Most researchers who do systematic reviews and meta-analyses rely on using information that has been published in the reports of trials. But these type of reviews can be limited, for example, if:

- many of the trials have not been published
- the reports are unclear
- the reports do not tell you all of the information that you are interested in

# What is an individual patient data meta-analysis?

This project (like most of the projects our group have done in the past) is called an individual patient data meta-analysis. The projects are carried out by an international Collaborative Group. This group is made up of:

- The Secretariat (members of the Meta-analysis Group)
- The Advisory Group (experts including doctors, statisticians and researchers)
- The Trial Investigators (the doctors who ran the trials)

At first, the Secretariat and Advisory Group members plan the project. We write a document (called the protocol) that describes why we think the project is important and what we plan to do. At the same time, we do a systematic review to find all of the trials that have been done.

Once the project is planned and we have found out about all of the trials, we can start to collect information for the meta-analysis. But, instead of using the information from the reports of these trials, we ask the Trial Investigators (the doctors who did the trials) to provide information on all of the patients in their trial (individual patient data). For example, we ask them to let us know the age of each patient, which treatment they received and what stage of cancer they had. Because we collect the individual patient data, we can use all of the trials, even those that have not been published or that had unclear reports or missing information. This type of meta-analysis is thought to be the best way of telling if one treatment works better than another.


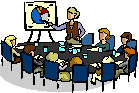
When we receive the individual patient data for a trial, we check that it makes sense. Once we have all the data from all the trials, we do our analyses and get the final meta-analysis results. We present these results first to the Collaborative Group. Then we write a report of the results, which get published in a medical journal.

# What do we already know about chemoradiation in cervical cancer?


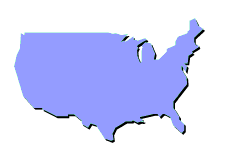


Many doctors are already using chemoradiation to treat women with cervical cancer. This is largely because in 1999, the National Cancer Institute in the USA released a statement to doctors. It said that doctors treating women for cervical cancer should consider using chemoradiation. The statement was made because the results of five randomised controlled trials suggested that in the trials women with cervical cancer who were treated with chemoradiation seemed to live for longer than those women who were treated with radiotherapy.


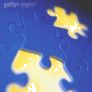
After the National Cancer Institute statement, a group of UK researchers did a systematic review and a meta-analysis based on the published reports of 19 randomised controlled trials. Their results also showed that chemoradiation might be a better treatment that radiotherapy alone. But, they could not get information from all of the trials because some had not been published or they were unclear or had missed out some important information.

So in this project, we have written to all of the doctors who did the trials and asked them to provide their individual patient data. We hope that by gathering together all of the information from all of the trials, we will be able to reliably answer many more questions about chemoradiation.

# What might this project tell us?

Although quite a lot of research has already been done to look at chemoradiation treatment for women with cervical cancer, we think that this project is really important. It may answer some questions that have not yet been answered.


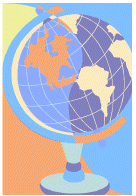
We have already done a systematic review. We have found 24 randomised controlled trials carried out all over the world including more than 5000 women. Together, these trials should help us get a more complete and reliable picture of the good and bad effects of chemoradiation.

We will ask questions like:

- Do women who receive chemoradiation live for longer than those who receive radiotherapy?
- Is chemoradiation better at stopping the cancer from coming back (recurring) than radiotherapy alone?
- Does chemoradiation reduce the chance of the cancer spreading to other parts of the body than radiotherapy alone?
- Do some women benefit more (or less?) from having chemoradiation than others?
- Does it matter what radiotherapy they have?
- Do some types of chemotherapy have a bigger effect than others?

We also want to look more closely at the side effects of chemoradiation. We have asked the doctors to include information for all women on a wide variety of possible side effects in the information that they send us. We can then try to properly answer questions like:

- - What are the short and long term side of chemoradiation?
    - How common are the side effects?
    - How severe are the side effects?
    - Are the side effects of chemoradiation worse than those of radiotherapy alone?
- Does anaemia affect how well chemoradiation works?

# Understanding the results of a meta-analysis

This section describes how we present the results of meta-analyses. It also talks about what the results mean. We won’t know the results of this meta-analysis until we have collected and checked the information from each of the trials and done the analyses. Therefore in this section we are using examples from other projects.

## What information do we collect on each woman?

Before we write to the doctors who ran the trials that we want to include, we think about what questions we would like to try to answer. These questions help us to decide what information (data) we will need to collect for each of the trials. For example, we want to find out whether chemoradiation stops the cancer from coming back and helps women to live for longer than women who had radiotherapy. So for each woman, we need to know:

- whether the woman was randomised to receive chemoradiation or radiotherapy
- what date they entered the trial
- whether or not the cancer has come back or spread
- whether or not the woman is alive
- either the date that they were last seen by the doctor or the date that they died

We would like to try to find out whether the effect of chemoradiation is different for different women. For example, do young or older women or women with different stages of cancer do better (or worse) with chemoradiation? For this, for each woman in the trial, we need to know:

- their age
- the stage of cancer they had

In this meta-analysis, we are also hoping to look at the side-effects that the women in the trials might have had because of their treatment for cervical cancer. So, we also asked the doctors for information on the side-effects that each women had, for example:

- If the woman had any nausea or vomiting, how bad was it (the grade of nausea / vomiting)?
- If the woman got anaemia during her treatment how bad was this (the grade of anaemia)

The information we collect for each woman in a trial is stored in a database, which ends up looking something like the example shown on the next page (Please note – this is not real data). Once we have collected in all of the information for every trial, we put all of the trial databases together in one large database and we use this for the analyses.

| **Patient ID** | **Trial ID** | **Age** | **Stage** | **Date of randomisation** | **Treatment Arm** | **Cancer come back?** | **Date cancer came back** | **Survival** | **Date of death / date last seen** | **Nausea during treatment (grade)** | **Anaemia during treatment  (grade)** |
| --- | --- | --- | --- | --- | --- | --- | --- | --- | --- | --- | --- |
| 0001 | 12 | 56 | 2a | 15/02/1989 | Radiotherapy | No | 22/09/2001 | Alive | 22/09/2001 | Grade 2 | None |
| 0002 | 12 | 49 | 3a | 19/05/1990 | Chemoradiation | No | 30/06/2002 | Alive | 30/06/2002 | Grade 1 | Grade 3 |
| 0003 | 12 | 62 | 2b | 26/09/1990 | Chemoradiation | No | 17/07/1998 | Dead | 04/06/2003 | Grade 2 | Grade 2 |
| 0004 | 12 | 46 | 2a | 08/11/1991 | Radiotherapy | Yes | 01/10/1993 | Alive | 11/12/1994 | Grade 3 | Unknown |
| 0005 | 12 | 51 | 3b | 19/12/1991 | Radiotherapy | Yes | 22/06/1995 | Dead | 27/11/1995 | Grade 1 | Grade 1 |
| 0006 | 12 | 67 | 3a | 05/02/1992 | Chemoradiation | No | 05/01/2002 | Alive | 23/04/2002 | None | Grade 2 |
| 0007 | 12 | 39 | 2a | 17/05/1992 | Chemoradiation | Yes | 19/12/2005 | Alive | 19/12/2005 | Grade 3 | Grade 1 |
| 0008 | 12 | 41 | 3b | 22/11/1992 | Radiotherapy | No | 02/02/2003 | Dead | 16/07/2003 | Grade 2 | None |
| 0009 | 12 | 68 | 2a | 17/02/1993 | Radiotherapy | No | 25/08/2001 | Alive | 25/08/2001 | Grade 2 | None |
| 0010 | 12 | 45 | 3a | 27/06/1993 | Chemoradiation | Yes | 15/03/2004 | Alive | 30/08/2004 | Grade 1 | Grade 3 |
| 0011 | 12 | 63 | 2b | 02/09/1993 | Chemoradiation | Yes | 16/11/2002 | Dead | 19/04/2003 | Grade 2 | Grade 2 |
| 0012 | 12 | 46 | 2a | 25/11/1993 | Radiotherapy | Yes | 21/10/2004 | Alive | 11/12/2004 | Grade 3 | Unknown |
| 0013 | 12 | 35 | 3b | 19/12/1993 | Radiotherapy | No | 27/02/1995 | Dead | 27/02/1995 | Grade 1 | Grade 1 |
| 0014 | 12 | 60 | 3a | 05/05/1994 | Chemoradiation | No | 26/09/2002 | Alive | 26/09/2002 | None | Grade 2 |
| 0015 | 12 | 42 | 2a | 17/08/1994 | Chemoradiation | No | 30/11/2004 | Alive | 31/05/2005 | Grade 3 | Grade 1 |
| 0016 | 12 | 41 | 3b | 17/10/1994 | Radiotherapy | Yes | 02/04/1999 | Dead | 09/09/1999 | Grade 2 | None |

An example of a trial database

## What data do we collect on the trial?

We also want to find out whether giving women a slightly different treatment (for example, different types of chemotherapy, or different doses of radiotherapy) makes a difference. So, we would ask the doctors to tell us:

- what drugs the women were given if they were randomised to receive chemoradiation
-
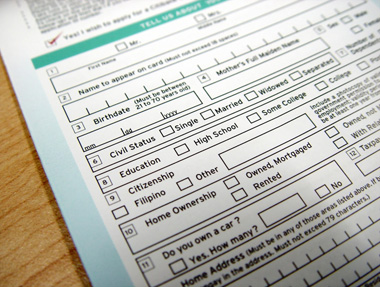
how long the course of radiotherapy lasted for

Usually, we collect this information on a form that we send out to each of the doctors who ran the trials. Once they return the form to us, we record the information in a table. It helps us to compare the trials with each other and is an easy way for us to check details about the trials when we need to during the project.

## How do we analyse the results?

When all the trials have been added to the final database, we can do the analyses. Usually in our meta-analyses, the result we would calculate is a Hazard Ratio (often abbreviated to HR). Hazard ratios are used when we are interested in when something happens as well as whether it happens. For example, when we are interested in the length of time a person is free of cancer. For hazard ratios, a value of 1 tells us that there is no difference between the two treatments. A value of less than 1 is where chemoradiation is better than radiotherapy, and a value of greater than 1 shows that radiotherapy is better than chemoradiation.

Sometimes we calculate an Odds Ratio (abbreviated to OR). This is usually when we are interested only in whether something has happened or not. Odds ratios are similar to hazard ratios, but a value of 0 tells us that there is no difference between the two treatments being compared. So in this meta-analysis, a value of less than 0 shows that chemoradiation is better than radiotherapy, and a value of greater than 0 shows that radiotherapy is better than chemoradiation.

First, the results of each trial are calculated. Then the results for the individual trials (the Hazard Ratios or Odds Ratios) are combined, using a statistical method, to get the overall result (again, a Hazard Ratio or Odds Ratio) for the meta-analysis. This meta-analysis result is a type of average of the results of all of the trials. So for example, if we are analysing the outcome of survival (“Is the survival of women receiving chemoradiation different to that of women receiving radiotherapy?”) and the hazard ratio = 0.80, we know (because 0.80 is less than 1) that survival is better amongst women receiving chemoradiation. The value of 0.80 (or 80%) tells us that there is a 20% improvement in survival with chemoradiation compared to radiotherapy.

## How do we display meta-analysis results?

The results of a meta-analysis are normally presented on a graph called a forest plot. An example forest plot is shown below.


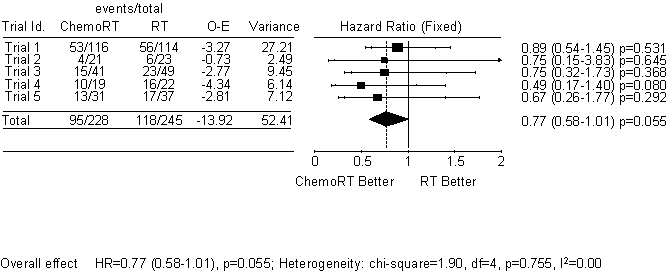


In the following sections, we will look at the different parts of the forest plot and explain what it all of the different parts means.

**1. The plot**

The right hand side of the forest plot depicts the results graphically. We will look at each part of the plot in turn.

i. The squares

The size of the square represents the amount of information that trial adds to the meta-analysis (or in simple terms, the size of the trial). A large trial with a lot of information is shown by a large square, smaller trials will be represented by smaller squares.

The position of the square for each trial represents the estimate of treatment effect for that trial. In this meta-analysis the most appropriate way to estimate the treatment effect (that is, the effect of chemoradiation compared with the effect of radiotherapy alone) is to calculate a statistic called the Hazard Ratio. Sometimes, meta-analyses will calculate other statistics.

1. The horizontal lines


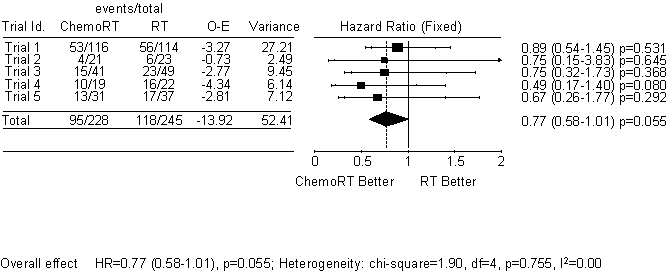


The horizontal line through either side of each square represents the “confidence interval” for each individual trial result. If a trial has narrow confidence intervals (shown by a short line) we can be more confident in the trial results. Where the trial has a big confidence interval (long line), we are less certain of the results of that trial.

1.
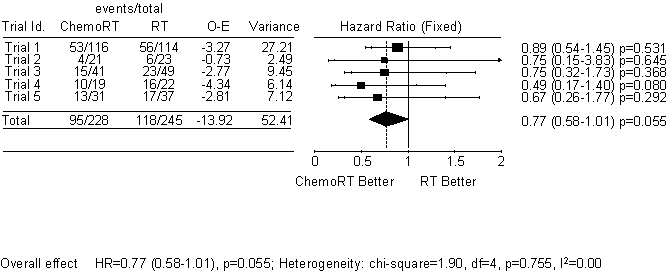
Vertical line through 1

Along the bottom of the plot, the line (or axis) goes from 0 to 2, with a vertical line passing through the value of 1. In a meta-analysis like this one, the value of 1 represents no difference between the two treatments. Values to the left of the line (or less than 1) represent trial results where chemoradiation is better than radiotherapy. Values to the right of the line (greater than 1) represent trials that have shown that radiotherapy is better than chemoradiation.

NB. On a plot that shows odds ratios (instead of hazard ratios) this line passes through the value 0. Values to the left of the line (less than 0) represent trial results where treatment is better than control. Values to the right of the line (greater than 0) represent trials that have shown that control is better than treatment.

1.
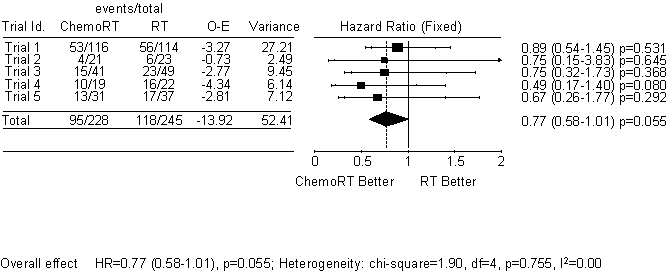
The diamond

At the bottom of the plot, the diamond shows the Hazard Ratio (HR) estimate of the effect of treatment overall for the meta-analysis. The centre of the diamond is positioned at the point of the estimate of the treatment effect. The edges of the diamond show the confidence intervals. Notice that the confidence intervals of a meta-analysis are narrower than those of the individual trials. This shows that the meta-analysis result is more reliable than any of the individual trials.

Some plots show results overall (for all of the trials grouped together) as well as for subsets (with only some of the trials grouped together). Usually, the diamonds for the subsets of trials are filled with stripes or dashes, and the overall diamond is solid.

For example, for a forest plot of survival of women who received chemoradiation compared with those who received radiotherapy, the table might show the numbers of women in each arm of each trial and the number of women who have died (the “events”).


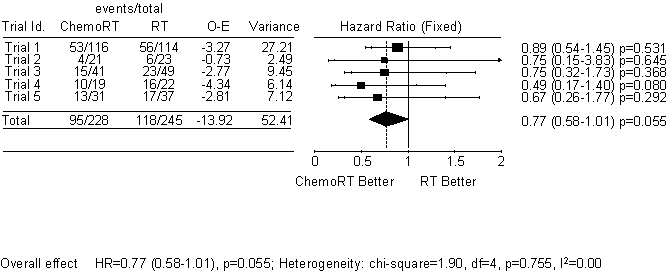
v. The numbers at the right of the plot

Sometimes, but not always, there will be some numbers at the far right of the plot, which line up with the squares for each trial and with the overall diamond for the meta-analysis results. The numbers shown above are highlighted as follows:

Yellow: hazard ratio estimates for each trial in the plot (see sections i. and iv. above)

Green: confidence intervals for the HRs for each trial (see sections ii. and iv. above)

Blue: p-values for the hazard ratio estimates for each trial (see section vi. below)

Red: overall hazard ratio estimate for the meta-analysis (see section iv. above)

Purple: confidence intervals for the overall hazard ratio for the meta- analysis (see section iv. above)

Grey: p-value for the hazard ratio for the meta-analysis (see section vi. below)

Sometimes all of these numbers will be shown on a plot, or else a combination of them will be shown (e.g. the HR estimates and p-values only).

vi. P-values

P-values are not represented on the plot itself, but are often shown in the numbers presented next to the plot (see section v. above). The p-value is a statistical term which indicates whether the result is “significant”. In general, if the results are significant (typically when the p-value is less than 0.05) then we can believe that there is a true difference between the two treatments being compared.


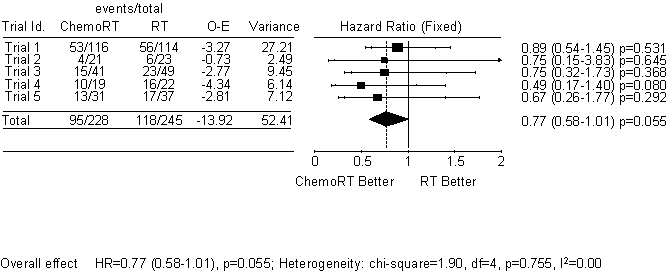
**2. Table of results**

The left hand side of the plot shows the results for each trial and the meta-analysis. This includes the number of patients and the ‘events’ on each arm of the trial. The term event means different things, depending on what we are analysing. For example, in an analysis of survival, an event will be recorded for each patient who has died. If we are looking at bone fractures, then an event would be recorded for every time a fracture was recorded.

In the table above, we can see that Trial 3 had 41 patients on the ChemoRT arm and there were 15 events. As this is the forest plot for an analysis of survival, this means that overall, there were 15 deaths recorded in the ChemoRT arm. The table also gives some other statistics that are used to calculate the estimates of the effect of the treatment. These statistics are calculated for each trial in the meta-analysis. The final row of the table shows the totals for each of the columns in the table. This is the meta-analysis totals, on which the results of the meta-analysis are based.

0

0.5

1

1.5

2

*Hazard Ratio*

**HR=0.86 (95% CI 0.77-0.95) p=0.003**

(no. events/no. entered)

CT

Control

O-E

Variance

Trial 1

43/82

41/71

-1.87

20.84

Trial 2

98/158

108/159

-13.61

51.00

Trial 3

53/102

60/104

-1.95

28.13

Trial 4

275/491

301/485

-23.69

143.61

Trial 5

68/151

84/160

-9.97

37.94

Trial 6

79/158

90/159

-6.37

42.18

Trial 7

70/78

60/75

1.79

31.96

**Total**

686/1220

744/1213

-55.67

355.65

Control better

Treatment better

## Example of a forest plot

In this example, it is clear to see that Trial 4 adds the most information to the meta-analysis (shown by the very large square). It also has the narrowest confidence intervals (shown by the short horizontal line through the square). The diamond showing the hazard ratio for the meta-analysis lies to the left of the line through 1, telling us that that the treatment is better than the control. The HR=0.86, which tells us that there is a 14% improvement with treatment compared to that with control. The confidence intervals around the overall HR (shown by the edges of the diamond) are narrow, so the result is quite reliable. The p-value is less than 0.05 and so we would say that the result is statistically significant. This means that we can be sure that there is a real difference between the effect of the treatment and the control.

# Being a Research Partner

## Why involve Research Partners?

In this research project, we would like to recruit women with personal experience of treatment with either radiotherapy or chemoradiation for cervical cancer to be Research Partners. We think that by working together with Research Partners on this project, we will able to:

- better reflect issues that may be important to women with cervical cancer
- identify important issues for women that have not been well researched and try to make sure that doctors’ think about these issues more carefully in their future research

We hope that the Research Partners enjoy their experience of being involved in and learning about the research that we do. We also hope that we can learn from the Research Partners and so improve this research project as well as those we do in the future. We have also set up a Reference Group to:

- help us to involve Research Partners
- advise us on how best to train and support Research Partners
- help us to explain the research project better
- let us know where we could be doing more

At the back of the folder are the Terms of Reference for the Research Partners. If you think that you might like to become a Research Partner, they should give you a better idea about the role of Research Partners in this project and to understand what we would expect from you as well as what you can expect from us.

If you are interested in being a research partner in this project, we would like to hear from you. Please contact us and we can give you more information on what this will involve.

# Conclusions


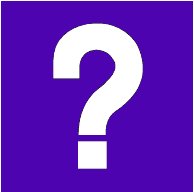
We hope that the information in this folder has helped you understand the project. However, if you have thought of questions to do with this project, you are welcome to get in touch with us. We will try to answer your questions or explain things that you are unsure of. At the back of this folder, there are some pages for your own notes and questions and a feedback form. We would be very grateful if you would fill this out and send it back to us. Your suggestions will help us to make the information better for this project and for future projects.

# Notes

Please use these pages for your own notes or to write down any questions that you would like to ask us.

**Notes**

# Feedback Form

We would very much appreciate your comments on the information in this folder. They will help us to improve it and will also help us in future projects. Please let us know what you think by returning your completed form in the stamped addressed envelope provided.

1. Is there any information in the folder that you do not want or need to know?

**Yes / No**

If YES, which parts do you think we should leave out? Please note them here, or highlight them in your copy of the folder.

2. Have we missed out anything that you would like to know about?

**Yes / No**

If YES, what other information would you like us to include?

3. Is the information clear?

**Yes /No**

4. Are there any words or sentences that you don’t understand?

**Yes / No**

If YES, please note them here or highlight them in your copy of the folder.

5. How would you describe the “tone of voice” in which the information in this folder is written?

6. Do you think it is the right “tone of voice”?

**Yes / No**

If NO, please say why you feel the tone of voice is wrong.

We would welcome any suggestions you may have on how we could improve the information in this folder, so please feel free to write additional comments on your copy.

**Thank you for taking the time to help us improve this information.**

# Research Partners: Draft Terms of Reference

The Terms of Reference should act as a guide for the Research Partners. They should give you a better idea about the your role in this project if you become a Research Partner. They explain what we would expect from you if you became a Research Partner, as well as what you can expect from us. The Terms of Reference can change in response to your feedback.

## Membership

We hope to set up a group of 5-6 Research Partners to be involved in the project. The two requirements that we think Research Partners need are:

1. Personal experience of either radiotherapy or chemoradiation treatment for cervical cancer
2. Interest and enthusiasm about being involved in this project

You do not need to have any previous experience of research. And, you are free to leave at any time, although we hope that all Research Partners would like to be involved throughout the project. We expect that this project will take around 2 years to complete.

## What we can provide to Research Partners

If you decide to become a Research Partner, we are committed to providing you with:

- An opportunity to learn about, actively contribute to and influence research
- Flexibility and choice in your level of involvement in the project
- A safe and supportive research environment
- Appropriate and relevant training
- A mentor, to act as your first point of contact for queries or difficulties
- Opportunities to meet other Research Partners to discuss the project
- Payment for attending meetings where needed

We will work with you to:

- Help you to learn about and understand the research project
- Identify research and/or administrative tasks that you can help with
- Provide access to appropriate training, where it might be helpful
- Support you in the activities that you undertake

We will provide a safe and supportive environment for your involvement, but the Research Partners group is not intended to be a Support Group. We are not able to give specific information or advice on treatments that you might have been given or may be receiving. However, we can put you in touch with groups or individuals who can provide emotional support and advice, should you want this.

## Key Responsibilities and Aims

As a Research Partner, you will work with each other and members of the Meta-analysis Group to carry out the research project. You will also feedback your thoughts and experiences of being involved in the project to the Meta-analysis Group and Reference Group. We would also like two Research Partners to be members of the project Reference Group. This responsibility could easily be shared between the Research Partners. There are separate Terms of Reference available for the Reference Group members.

There are many aspects to running a research project like this one. Some of these are administrative or creative and not necessarily scientific or technical. We can discuss the possibilities with you to help you to find things that interest you and that you might like to be involved with. For example there may be opportunities to:

- Read and comment on the information that we produce about the project for women who have had or have cervical cancer
- Help us to organise small local meetings or the large Collaborators’ meeting at the end of the project
- Write short articles for the newsletter
- Help to produce and disseminate the results of the project to women who have had or have cervical cancer

You can be involved in one or more of the many aspects of this project. What you do might change as the project progresses. For example, once you have a clearer idea of the project, and have maybe taken up opportunities for training, you might take on different tasks or suggest other areas where your skills could put to good use. We will work with you to develop these opportunities.

## Meetings and Communications

We can arrange to visit you or a have a telephone conversation with you so that you can find out more about project before you decide whether or not to become a Research Partner. Once 5 or 6 women have agreed to be Research Partners, we will organise a meeting at the MRC Clinical Trials Unit, or at another convenient location. We will try to keep this meeting (and any additional meetings we have) very informal and we won’t use technical language or jargon. This meeting will give you:

- Chance to meet the other Research Partners
- Chance to meet the Meta-analysis Group members who are running this project
- Opportunity to find out more about the project and decide if you would like to be involved in it

After this first meeting, we will contact you in the way you prefer, and we can send you information in whichever format suits you best (e.g. on paper, email, large print etc). If you need to ask any questions or if you want us to explain something, you can contact us during our working hours to ask. We will do our best to answer all enquiries promptly and clearly. We might also organise other meetings of the Research Partners during the project, but we would like to keep the number of meetings to a minimum. We will try to arrange them to suit the needs of the individuals.

## Payment

Where needed, we can reimburse you for travel or other expenses for attending meetings. These might include overnight accommodation or carer costs, for example. Wherever possible, we will try to pay for all travel tickets and accommodation bookings in advance. Otherwise, we will aim to reimburse expenses as quickly as possible. In addition, we are able to pay Research Partners for their time at the standard Department of Health rate (currently £138.71/day).

## Person Specification

| **Attributes** | **Essential** | **Desirable** | **Not  important** |
| --- | --- | --- | --- |
| Enthusiastic about the project | ✓ |  |  |
| Committee Experience |  |  | ✓ |
| Aware of issues that might affect women with cervical cancer |  | ✓ |  |
| Understanding of medical / research language |  |  | ✓ |
| Experience of chemoradiation or radiotherapy treatment for cervical cancer | ✓ |  |  |
| Good communicator /able to express own views in a mixed group of professionals and consumers |  | ✓ |  |

# Glossary

**Adenocarcinoma**A type of cancer that starts in gland cells. Adenocarcinoma of the cervix starts in gland cells that are found in the passageway from the cervix to the womb

**Adjuvant**

An adjuvant treatment is one which is given after the main treatment. For example, adjuvant chemotherapy is often given to patients who have had an operation to remove a cancer.

**Brachytherapy**

Brachytherapy is radiotherapy that is given from inside the body. In cervical cancer, this is done by putting a radioactive source into the vagina and womb. It is also sometimes called internal radiotherapy or intracavitary radiotherapy.

**Chemoradiation**

Chemoradiation is where chemotherapy and radiotherapy are given to a patient at the same time. It is thought that giving chemotherapy at the same time as radiotherapy makes some types of cancer more sensitive to radiotherapy.

**Chemotherapy**

Chemotherapy is the term used for any type of drug treatment that is used to try to kill cancer cells. In cervical cancer, chemotherapy might be used along with either surgery or radiotherapy, to make sure that all of the cancer cells have been killed and to prevent the cancer from coming back.

**Clinical trials**Clinical trials are research studies involving patients, which compare a new or different type of treatment with the best treatment currently available.  They test whether the new or different treatment is safe, effective and any better than what already exists.  No matter how promising a new treatment may appear during tests in a laboratory, it must go through clinical trials before its benefits and risks can really be known.

**Forest plot**

A forest plot is the typical type of plot or figure used in a meta-analysis. It shows the results (e.g. hazard ratios or odds ratios) for all of the trial, groups of trials or overall meta-analysis in the form of a graph. It also represents other statistical information relating to the results.

**Hazard ratio**

A hazard ratio (or HR) is the statistic used to give the results of randomised controlled trials or meta-analyses. Hazard ratios are used when we are interested in when something happens as well as whether it happens. For example, when we are interested in the length of time a person is free of cancer. For hazard ratios, a value of 1 tells us that there is no difference between the two treatments. A value of less than 1 is where treatment is better than control, and a value of greater than 1 shows that the control treatment is better.

**Meta-analysis**A meta-analysis involves a researcher bringing together the numerical results of previous research (usually randomised trials) about one particular treatment.

A meta-analysis can be important because it allows us to pick up small differences between treatments.  These differences can be very hard to spot, so trials need to include large numbers of patients to pick these up.  Many trials are not big enough, so we cannot be sure whether any differences that we find are because of differences between the treatments or just due to chance.  By bringing together the results of all trials of a particular treatment in a meta- analysis, we can look at the experience of many more patients.  This gives a more reliable and accurate measurement of the effect of the treatment and a good chance of seeing which treatments are best.

In an individual patient data (IPD) meta-analysis, the researchers go back and look at the records for each patient who took part in a trial (these are anonymised, so the researchers don’t know patients’ names).  Then they bring these individual results together.  This makes the results of the meta-analysis more reliable and enables researchers to look at how treatments have worked in different groups of patients, e.g. age group or sex.

**Neoadjuvant**

Neoadjuvant treatments are given before the main treatment. For example, often chemotherapy is given before an operation to remove a cancer, or before radiotherapy.

**Odds ratio**

An odds ratio (or OR) is a statistic used to give the results of randomised controlled trials or meta-analyses. This is usually when we are interested only in whether something has happened or not (and not when it happened). Odds ratios are similar to hazard ratios, but a value of 0 tells us that there is no difference between the two treatments being compared. A value of less than 0 shows that treatment is better than copntrol, and a value of greater than 0 shows that the control is better.

**Protocol**

A protocol is the plan for a piece of research.  It usually includes information about:

- What question the research is asking and its importance/relevance
- The background and context of the research, including what other research has been done before
- How many people will be involved
- Who can take part
- The research method
- What will happen to the results and how they will be publicised

A protocol describes in great detail what the researchers will do during the research.  Usually, it cannot be changed without going back to a research ethics committee for approval.

**Radiotherapy**

Radiotherapy is where x-rays are used to try to kill the cancer cells. Radiotherapy can be given from outside the body (external radiotherapy) or from inside the body (internal radiotherapy or brachytherapy).

**Randomisation**

If you take part in a randomised controlled trial, you will have an equal chance of receiving any of the treatments being compared.  The decision about which treatment you’ll receive is random – or based on chance.  A computer will decide which treatment you’ll receive, not you or the doctor.  This is called randomisation.

Randomisation ensures that the two groups of people in a trial are as similar as possible, except for the treatment they receive.  This is important because it means that researchers can be sure that any differences between the groups are only due to the treatment.

Randomisation is also the best way of ensuring that the results of trials are not biased. For example, if a doctor chose which treatment a patient should receive as part of a trial, she or he might give the new treatment to sicker patients, or to younger patients.  This would make the results of a trial unreliable.  Randomisation helps prevent this kind of bias.

**Randomised controlled trial**

Many clinical trials are randomised controlled trials (RCTs).  Clinical trials aim to make a fair comparison between a new treatment and the current treatment on offer, or between two (or more) existing treatments, to see which one works best.

A controlled trial compares two groups of people: an experimental group who receive the new treatment and a control group, who receive the usual treatment or a placebo.  The control group allows the researchers to see whether the treatment they are testing is any more or less effective than the usual or standard treatment.

If you take part in a randomised controlled trial, you will have an equal chance of receiving any of the treatments being compared.  The decision about which treatment you’ll receive is random – or based on chance.  A computer will decide which treatment you’ll receive, not you or the doctor.  This is called randomisation.

Randomisation ensures that the two groups of people in a trial are as similar as possible, except for the treatment they receive.  This is important because it means that researchers can be sure that any differences between the groups are only due to the treatment.

Randomisation is also the best way of ensuring that the results of trials are not biased.  For example, if a doctor chose which treatment a patient should receive as part of a trial, she or he might give the new treatment to sicker patients, or to younger patients.  This would make the results of a trial unreliable.  Randomisation helps prevent this kind of bias.

**Side effects**

Side effects, or adverse reactions, are undesired effects of a treatment.  For example, if you are given a drug to treat a mental illness and it makes you sick, the sickness would be described as a side effect.  Clinical trials will often look at the short-term and long-term side effects of a treatment.

**Squamous cell carcinoma**

Squamous cell carcinoma is a type of cancer that starts in skin-like cells. Squamous cell carcinoma of the cervix starts in the skin-like cells that cover the outside of the cervix.

**Stage**The stage of a cancer describes how big the tumour is and how far it has spread.  Doctors use a system for cervical cancer that numbers the different stages from 0 (smallest tumours with least spread) to 4 (Largest tumours/ tumours that have spread furthest).

**Surgery**
Surgery is the term used for any operation. There are different types of surgery (operations) that can be used to treat women with cervical cancer. The type of surgery used will depend on factors such as the size or the stage of the cancer.

**Systematic review**

Systematic reviews aim to bring together the results of all studies addressing a particular research question that has been carried out around the world.  They provide a comprehensive and unbiased summary of the research.

For example, one clinical trial may not give a clear answer about the effectiveness of a treatment.  This might be because the difference between the treatments being tested was very small, or because only a small number of people took part in the trial.  So systematic reviews are used to bring the results of a number of similar trials together, to piece together and assess the quality of all of the evidence.  Combining the results from a number of trials may give a clearer picture.  When researchers look at the numerical results of these trials and compare them, this is called a meta-analysis.
